# Fugulin scale for classifying pediatric patients in a respiratory inpatient unit: experience report

**DOI:** 10.1590/1980-220X-REEUSP-2022-0454en

**Published:** 2023-09-15

**Authors:** Mitzy Tannia Reichembach Danski, Letícia Pontes, Izabela Linha Secco, Higor Pacheco Pereira, Solange Cristina Moreira Vieira, Elisangela Dalmaz Freitas, Juliana Szreider de Azevedo, Regiane Queiroz Afonso

**Affiliations:** 1 Universidade Federal do Paraná, Programa de Pós-Graduação em Enfermagem, Curitiba, PR, Brazil.; 2 Hospital Infantil Doutor Waldemar Monastier, Campo Largo, PR, Brazil.

**Keywords:** Classification, Severe Acute Respiratory Syndrome, Pediatric Nursing, Nursing Staff, Hospital, Patient safety, Clasificación, Síndrome Respiratorio Agudo Grave, Enfermería Pediátrica, Personal de Enfermería en Hospital, Seguridad del paciente, Classificação, Síndrome Respiratória Aguda Grave, Enfermagem Pediátrica, Recursos Humanos de Enfermagem no Hospital, Segurança do Paciente

## Abstract

**Objective:**

To describe the use of the Fugulin scale to classify pediatric patients hospitalized in a respiratory unit as a subsidy for the allocation of human resources given the increase in cases of Severe Acute Respiratory Syndrome.

**Method:**

Experience report conducted in a children’s hospital in the Metropolitan Region of Curitiba with data collection from medical records and approved by the Institution and by the Research Ethics Committee.

**Results:**

Between February and May 2022, the percentage of patients categorized in minimal and intermediate care decreased by 53 and 11.4%, respectively, while those in high dependency and semi-intensive care expanded by 31.2 and 84.2%. In addition, in just four months, there was a considerable increase in the positivity of virologies compared to the twelve months of 2021. The susceptibility of children to the development of severe respiratory infection was proven through the decrease in virologies with undetectable results.

**Conclusion:**

The results obtained allowed us to conclude there was a significant increase in the complexity of patients admitted to the respiratory unit, showing it is essential to provide a nursing team compatible with the care needs.

## RESUMO

**Objetivo:**

Descrever sobre a utilização da escala de Fugulin para classificação de pacientes pediátricos internados em uma unidade respiratória como subsídio para a alocação de recursos humanos, diante do aumento de casos de Síndrome Respiratória Aguda Grave.

**Método:**

Relato de experiência com coleta de dados em prontuário, realizado em um hospital infantil da região metropolitana de Curitiba, aprovado pela Instituição e pelo Comitê de Ética em Pesquisa com Seres Humanos.

**Resultados:**

Entre fevereiro a maio de 2022, a porcentagem dos pacientes categorizados em cuidados mínimos e intermediários decaiu em 53 e 11,4%, respectivamente, ao passo que os de cuidados de alta dependência e semi-intensivos expandiram 31,2 e 84,2%. Além disso, em apenas quatro meses, houve aumento considerável na positividade das virologias em comparação aos doze meses de 2021. A suscetibilidade das crianças ao desenvolvimento de infecção respiratória grave foi comprovada através da diminuição de virologias com resultados não detectáveis.

**Conclusão:**

Os resultados obtidos permitiram concluir que houve um aumento expressivo da complexidade dos pacientes admitidos na unidade respiratória, sendo essencial o provimento de equipe de enfermagem compatível com as necessidades de cuidados.

## RESUMEN

**Objetivo:**

Describir el uso de la escala de Fugulin para la clasificación de pacientes pediátricos hospitalizados en una unidad respiratoria como subsidio para la asignación de recursos humanos, ante el aumento de casos de Síndrome Respiratorio Agudo Grave.

**Método:**

Relato de experiencia con recolección de datos de registros médicos, realizado en un hospital infantil de la región metropolitana de Curitiba, aprobado por la Institución y por el Comité de Ética en Investigación con Seres Humanos.

**Resultados:**

Entre febrero y mayo de 2022, el porcentaje de pacientes categorizados en cuidados mínimos e intermedios disminuyó en 53 y 11,4%, respectivamente, mientras que los de alta dependencia y cuidados semiintensivos se expandieron a un 31,2 y un 84,2%. Además, en tan solo cuatro meses se registró un aumento considerable de la positividad de virologías respecto a los doce meses de 2021. Se comprobó la susceptibilidad de los niños al desarrollo de infección respiratoria grave a través de la disminución de virologías con resultados indetectables.

**Conclusión:**

Los resultados obtenidos permitieron concluir que hubo un aumento significativo en la complejidad de los pacientes ingresados en la unidad respiratoria, demostrando que es fundamental disponer de un equipo de enfermería compatible con las necesidades asistenciales.

## INTRODUCTION

As the pandemic associated with the SARS-CoV-2 broke in Brazil, it was observed that children did not seem to be responsible for a large proportion of infections caused by this virus, and early evidence supported that most children them did not present the severe disease^(1)^.

Although the clinical impact in the first pandemic year was not as exuberant in pediatrics compared to adults, in 2022 there was evidence of an early increase in cases of respiratory infections in children caused by other etiological agents, such as the Influenza virus, Respiratory Syncytial, Adenovirus, Rhinovirus and Metapneumovirus, which spread more according to their seasonality in the southern region of Brazil (April-August)^(2)^. The Oswaldo Cruz Foundation (Fiocruz) InfoGripe Bulletin issued in April this year signaled that the incidence of Severe Acute Respiratory Syndrome (SARS) in children had already shown a sign of significant rise in several Brazilian states since February, reaching a peak of up to 309% compared to the previous year^(3)^.

The main characteristic of patients with severe respiratory disease is the development of SARS, defined by the acute onset of hypoxemic-type respiratory failure with bilateral pulmonary infiltrates. Cases of flu-like illness that present with respiratory distress and/or hypoxia defined as SARS must be treated in hospitalization, and indications for the intensive care sector include respiratory failure, presence of shock and/or organic dysfunction or, after cardiac arrest event. Therefore, early identification of the disease, immediate intervention and installation of adequate monitoring are important^(1)^. Likewise, the adequacy of the nursing team is essential, considering that the quantity and quality of these professionals directly interfere with the safety and quality of patient care^(4)^.

The Federal Council of Nursing (Portuguese acronym: COFEN), based on Resolution number 543/2017, determines that it is the nurse’s responsibility to establish the quantitative and qualitative structure of professionals necessary to provide nursing care in order to achieve the standard of excellence in care and favor not only patient safety, but that of the health team and institution. In this context, the dimensioning of nursing staff should be based on characteristics related to the health service, the nursing service and the patient, the latter being based on the degree of dependency and formally evaluated through the Patient Classification System (PCS)^(4)^.

In pediatrics, there are some assessment scales that help nurses in the PCS, such as the Pediatric Patient Classification Instrument^(5)^. However, they are not always easily available in practice, being underused. The Fugulin scale, although unspecific for an age group, is an option for children and allows categorizing the degree of dependency of patients in minimal care (12–17 points), intermediate (18–22 points), high dependency (23–28 points), semi-intensive (29–34 points) and intensive (34–48 points), through nine areas of care: mental status, oxygenation, vital signs, nutrition, mobility, walking, bodily care, elimination and therapy^(6)^. It was developed and implemented 14 years ago in a hospital in southeastern Brazil^(7)^, endorsed by COFEN Resolution number 543/2017^(4)^.

In view of the above, the purpose of this experience report is to describe the use of the Fugulin scale to classify pediatric patients hospitalized in a respiratory unit as a support for the allocation of human resources, given the increase in cases of SARS.

## METHOD

### Study Design

The Standards for Reporting Qualitative Research (SRQR) was the tool adopted for the development of this report^(8)^.

Experience reports do not come from research. They aim to describe the individual experience or that of a group of professionals on a given situation. Even thoughthere is still no specific guideline to guide the writing of experience report articles, the use of the SRQR is suggested^(9)^.

### Location

The present report took place in a children’s hospital in the Metropolitan Region of Curitiba. This state public institution serves the vast majority of the child population of Curitiba and the Metropolitan Region, in the age group from zero to 17 years. The hospital also assists children and adolescents from other cities in the state of Paraná and, to a lesser extent, from other states.

Inaugurated on December 14, 2009, the hospital serves medium and high complexity patients, currently providing 89 active beds: 30 critical unit beds - 20 neonatal and 10 pediatric, 35 clinical ward beds and 24 surgical ward beds. It also has a surgical center with three rooms in operation and an outpatient clinic for pediatric medical specialties (neurology, neurosurgery, high-risk childcare, nephrology, pulmonology, medical nutrition therapy, ophthalmology, cardiology, dermatology, otorhinolaryngology, gastroenterology, orthopedics, vascular surgery, plastic surgery and pediatric surgery).

The clinical ward admits patients referred by the State Bed Regulation Center, and they are assisted by a complete multidisciplinary team. For a total of 35 beds, there are two nurses, seven nursing technicians, three doctors (two routine and one on duty) and a physiotherapist. The other specialties, such as psychology, social work, occupational therapy, nutrition and speech therapy, are part of the interdisciplinary team and hired on demand (per day).

According to data provided by the Management Information System of the hospital, the occupancy rate in that unit during the study period ranged from 84% in February, 97% in March, to 98% in April, reaching a maximum peak of 100% in May, which corresponded to the second month of seasonality of respiratory viruses in the southern region of the country. The clinical ward became a respiratory unit to absorb the exponential increase in respiratory cases in Paraná, with an emergency expansion of another 10 beds (previously there were 25) and readjustment of institutional protocols to assist referred patients.

### Population and Selection Criteria

As per the routine already established at the institution, every patient admitted to the clinical ward is classified according to complexity using the Fugulin scale, which is computerized in an electronic medical record. After admission, the PCS is redone every 24 hours until hospital discharge.

### Data Collection

**Figure 1:**
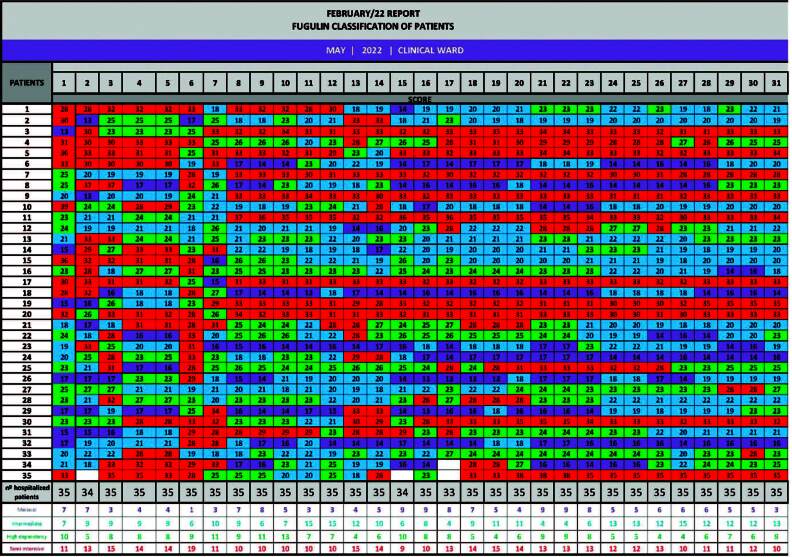
Data collection instrument filled in with information collected in May/2022.

Data were collected from the electronic medical records of patients hospitalized between February and May 2022. As the application of the Fugulin scale is already part of the routine of care nurses at the unit, the authors extracted information on the degree of dependency of patients every 24 hours, always retroactively (referring to the previous day). A collection instrument was prepared in a table format in Excel^®^, divided into 30-31 columns representing the days of the month and 35 lines for each active hospital bed. It is presented in Figure 1, filled in with data referring to the month of May.

According to previous organization, each author checked how many patients had been hospitalized in the previous 24 hours in the unit, extracted the Fugulin scale value for each one and colored the cell according to the following legend: minimal care (purple), intermediate care (blue), high dependency care (green) and semi-intensive care (red). At the end of the 35^th^ bed computed, the total number of patients admitted that day was described, in addition to the sum of how many patients fit into each level of dependency. In the first week following the end of the month, the authors met to analyze and process the collected data.

In addition to collecting data from medical records to classify patients according to complexity, data regarding the results of virology during the study period were recorded - laboratory tests that investigate the causal agent of respiratory infection, covering seven types of viruses: Influenza A and B, SARS- CoV-2, Adenovirus, Respiratory Syncytial Virus, Metapneumovirus and Rhinovirus. This exam is collected at the institution, but forwarded to the State Central Laboratory (Portuguese acronym: LACEN) to be processed. Monitoring until issuance of the report is carried out daily by Hospital Infection Control (Portuguese acronym: CCIH), through a nationwide platform known as Laboratory Environment Manager. Based on the result, the care teams are notified and the CCIH feeds an Excel^®^ spreadsheet, from where information regarding virology was extracted and analyzed.

### Data Analysis and Processing

After monthly data tabulation, the research team analyzed the information obtained through descriptive statistics and development of graphics. Some variables could be compared with the year 2021 and helped in the interpretation of some outcomes.

### Ethical Aspects

This experience report is part of a structuring project approved by the Research Ethics Committee of the Complexo Hospital de Clínicas – Universidade Federal do Paraná, according to opinion number 2.947.877 of 10/08/2018, respecting the ethical precepts of resolution 466/2012. As data were collected from medical records, the informed consent form was not necessary.

## RESULTS

In view of the exponential increase in SARS cases in the pediatric population, from February 2022 the clinical ward was reformulated to meet this demand, becoming exclusively a respiratory unit. Senior management restructured flows and guidelines for care, but it was also necessary to develop a strategy to justify the allocation of human resources, since there is no specific legislation for hospitalization sectors that determines the number of nursing staff per available beds.

In addition to the immediate need to expand the service, as the collection instrument was being completed, it was observed that the complexity of patients also increased (Table 1). The percentage of patients categorized into minimal and intermediate care declined by 53 and 11.4%, respectively, while high dependency and semi-intensive care expanded 31.2% and 84.2%, respectively.

**Table 1:** Percentage of evolution of the complexity of hospitalized patients over the study period – Campo Largo, PR, Brazil, 2022.

Complexity	February/2022	March/2022	April/2022	May/2022
Minimal care	34%	33%	15%	16%
Intermediate care	32%	27%	26%	28%
High dependency care	16%	18%	27%	21%
Semi-intensive care	19%	23%	33%	35%

A comparative analysis corresponding to the twelve months of 2021 and February-May 2022 proved to be important as it also showed an increase in the number of respiratory conditions by type of microorganism. Note that despite the disproportionate comparison between years, the year 2022 greatly surpassed year 2021 statistics in just four months. The differences between the number of cases are described in Table 2.

**Table 2:** Number of respiratory cases by type of microorganism between 2021 and 2022 – Campo Largo, PR, Brazil, 2022.

Microorganism	2021 (January–December)	2022 (February–May)
Rhinovirus	59	69
Metapneumovirus	0	36
SARS-CoV-2	25	58
Undetectable	142	51
Respiratory Syncytial	52	95
Adenovirus	3	13
Influenza A	0	8
Total	281	330

**Figure 2:**
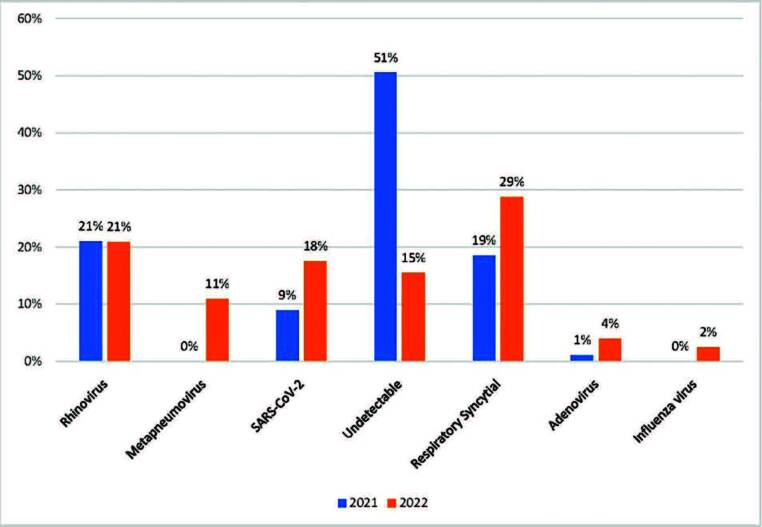
Comparison between the positivity of virology tests and the type of microorganism between 2021 and 2022.

From the analysis above, another notable outcome was the reduction in cases in which the pathogen was not detected by the virology test (Figure 2).

## DISCUSSION

The findings related to the demand for care through the Fugulin scale revealed there was a significant increase in the complexity of patients hospitalized in the respiratory ward during the study period. Such information suggests a reflection on the profile of patients hospitalized in inpatient units, which require high availability of care from the nursing team^(10)^, a scenario that is often underestimated by hospital managers because there is no specific regulation designating the appropriate quantity of the category in these sectors.

Inpatient units can admit patients with different levels of clinical complexity, promoting situations of risk and vulnerability for the patient. This scenario exerts direct impact on care needs and requires a vigilant nursing team^(11)^.

It is important to emphasize that the provision of care in pediatrics differs from that in other age groups in some aspects, as it is not restricted to direct care for the child. It demands guidance and health education, as well as emotional support for companions. As nursing is the professional category closest to the patient, during the hospitalization period they need to have time within their work routine for these issues that go beyond the care practice^(10)^.

Considering the high workload in these units, “one of the challenges for managers is to properly dimension the team in order to provide safe and evidence-based patient care. However, it is still notorious that health institutions plan personnel costs based on limited financial resources”^(12)^.

Personnel sizing is a methodological process that allows calculating the number of nursing professionals needed to care for patients in accordance with their degree of dependency. One of the most relevant variables for its measurement is the nursing workload, which can be obtained by analyzing the total hours of care. The PCS, which stipulates the hours of care according to the care complexity of the patient, has been used in Brazil^(12)^.

Although it is considered an indirect tool for the PCS, categorizing the patient by the degree of dependency constitutes the first step in calculating the sizing. The daily and systematic classification not only allows an adequate organization of care and professional quantity, but also means a reorientation towards user-centered nursing care, articulating improvements for the creation of favorable and positive practice environments^(13)^.

Before the collections started, the research team found it difficult to sensitize clinical nurses about the need to apply the Fugulin tool. Resistance was justified by the lack of specificity of this scale for pediatrics. Therefore, one of the measures to break this paradigm was to equate the clinical variables to the institutional reality, standardizing the interpretation of each one of them by nurses so that the results expressed the degree of dependency reliably. Any instrument can be restructured according to the reality of care needs, the specific characteristics of patients and operational issues. A PCS can be adapted to each institution according to the characteristics of the service, even validated, as found in a study with pediatric patients hospitalized in a high complexity sector^(5)^. Currently, all patients admitted to the respiratory ward continue to be categorized every 24 hours according to the Fugulin scale, demonstrating nurses’ understanding of its application.

Other reflections also helped nurses to recognize such importance in the context of respiratory infections in order to mobilize the hiring and sufficient number of nursing professionals. There was also an indirect pandemic impact on child’s health related to school closures, developmental delay, outdated immunization schedule, increased sedentary lifestyle, and food precariousness in this population^(14)^. Therefore, it can be inferred that one of the main reasons for the increase in SARS cases and complexity in 2022 compared to 2021 was social isolation, because when children and adolescents returned to their activities at the beginning of that year, there was a background of delayed immunity.

The evidence in this study that corroborates the issue of immunity was the decrease in undetectable SARS cases in 2022. As soon as children and adolescents returned to school, exposure to respiratory viruses associated with delayed immune defense increased the chance of developing a more severe respiratory condition. Therefore, a reduction in cases of SARS not confirmed by laboratory was observed, indicating that since there was contact between the virus and the host, susceptibility to infection was greater in 2022.

Although SARS etiology is associated with more than one virus, immunization against one of its etiological agents, SARS-CoV-2, reduces the complexity of the disease. The Brazilian Societies of Pediatrics and Immunizations jointly published their position in favor of vaccinating children against COVID-19, in the light of robust scientific evidence now in force,from the beginning of vaccination in the age group of 5 to 11 years, which began in January 2022^(15)^.

Paraphrasing this document, the Ministry of Health published in its Epidemiological Bulletins that the burden of the disease in the Brazilian population of children is relevant. Until October 2022, 12,634 hospitalizations by SARS resulting from COVID-19 were registered, with 463 confirmed deaths in children aged up to five years. Note that the use of vaccines in the adult and adolescent population with high vaccination coverage in Brazil and in several countries around the world leads toa considerable increase in the relative number of infected children^(15)^.

Currently, with the circulation of variants and their several subvariants in the country, this situation remains, with a higher risk of hospitalizations, complications and deaths in people who are not vaccinated or have incomplete regimens. Likewise, an increase in the risk of severe forms of the disease, such as long-term COVID-19 and multisystem inflammatory syndrome (MIS-C) is observed among children, and these are non-negligible complications in this population^(15)^. A well-designed study involving 4,516 children aged between six months and four years inferred that the vaccine against SARS-CoV-2 is safe and immunogenic, that is, it causes a satisfactory immune response^(15)^.

As much as experts defend the COVID-19 vaccination scheme for children, a national survey conducted by Fiocruz with parents of this target audience pointed to hesitation by 16.4% of parents of children aged 0-4 years, 12.8% of parents of children aged 5-11 years, and 14.9% of parents of teenagers. Although hesitant parents are a minority, one of the beliefs associated with the high percentage of insecurity was the fear of an adverse reaction^(16)^. Therefore, the increased complexity of SARS cases triggered by SARS-CoV-2 may be associated with parents’ non-adherence to the vaccination of their children.

Faced with a context of such clinical relevance, a very important issue is also structuring the care of these children and adolescents at the hospital level. The lack of use of a PCS as a management method in nursing can generate work overload, compromise the care practice, increase morbidity and mortality rates, length of stay and, consequently, hospital costs. Furthermore, it influences the risk of emotional exhaustion, stress, dissatisfaction at work and burnout in the nursing team, reflecting on absenteeism and turnover rates^(6)^.

Based on the existing relationship between the workforce and the quality/safety of health services, research has been conducted with the aim to evaluate this cause and effect link. A cross-sectional study conducted in 243 European hospitals measured the following outcome variables: mortality, patient complexity, quality of care, safety, adverse events, burnout and job dissatisfaction. The main conclusions were that the increase in the percentage of nurses was associated with lower chances of death, of patients with complex clinical conditions, and of poor quality of care. On the other hand, for every 10% reduction in the number of these professionals, the risk of death among patients increased by 11%^(17)^.

Another cross-sectional study proposed to examine the effects of nurses’ occupational fatigue through care left undone. The average number of tasks not performed was 3.45 (SD = 2.19). The higher the levels of fatigue the greater the occurrences of care left undone, and the opposite was also proven. The main clinical relevance of this Korean study was to demonstrate to health administrators and leaders how crucial it is to develop tools to reduce excessive workloads and provide patient safety^(18)^. A systematic review with a similar objective concluded that low hiring of nursing staff is associated with reports of omissions of care in hospitals, where missed care becomes a promising indicator of nurse staffing adequacy^(19)^.

In the Brazilian scenario, authors identified that the main reasons for omissions of care were labor resources and the inadequate number of personnel. A significant and inversely proportional correlation was found between patient safety culture and omissions in nursing care (r = –0.393)^(20)^. In pediatric hospitals, the factors that influence missed nursing care are also in line with international studies, prolonging the hospitalization period^(21)^.

Finally, the need to classify the degree of dependency of patients has become a priority, as with these data it is possible to predict and ensure the adequate number of nursing professionals to provide care. As a high-quality management tool, staff dimensioning using the Fugulin scale suited to the uniqueness of the health service ensures safety for users and workers^(6)^.

Based on the assumption that the Fugulin scale was validated in Brazil by a nurse, the main limitation of this study is related to the scarcity of national and current scientific publications on its application in pediatrics in order to discuss the agreements and disagreements with other already published studies. Furthermore, most studies on PCS focus on intensive care units, with little emphasis on inpatient units.

Therefore, this case report intends to contribute with nurses and health managers so they can provide human resources based on scientific evidence, praising the use of an exclusively Brazilian scale in a pediatric reality in the country, which included ward patients.

## CONCLUSION

The results obtained were able to identify the care profile of children and adolescents treated at the respiratory unit. They allowed us to conclude there was a significant statistical increase in the complexity of pediatric patients. Based on this outcome, it was possible to guarantee the quality and safety of nursing care with the provision of a team compatible with the care needs.

The scientific framework built over three years of the pandemic to guide clinical practice in SARS cases has become increasingly precise, with high levels of evidence. However, preventive measures are equally important in controlling SARS, regardless of the etiologic agent. Among the main recommendations to avoid contagion and reinfections by respiratory viruses, we highlight the complete vaccination coverage of the population, including booster doses, hand hygiene at opportune times, promotion of more ventilated and cleaner environments and the proper use of the mask in crowded places.
