# ERRATUM: Fugulin scale for classifying pediatric patients in a respiratory inpatient unit: experience report

**DOI:** 10.1590/1980-220X-REEUSP-2022-0454eren

**Published:** 2024-02-23

In the article “Fugulin scale for classifying pediatric patients in a respiratory inpatient unit: experience report”, with DOI code number: https://doi.org/10.1590/1980-220X-REEUSP-2022-0454en, published at Revista da Escola de Enfermagem da USP [online], v. 57: e20220454, on the page 6:

**Where it was written:**

**DESCRITORES**

Classificação; Síndrome Respiratória Aguda Grave; Enfermagem Pediátrica; Personal de Enfermería en Hospital; Segurança do Paciente.

**DESCRIPTORES**

Clasificación; Síndrome Respiratorio Agudo Grave; Enfermería Pediátrica; Recursos Humanos de Enfermería en el Hospital; Seguridad del paciente.

**Should read:**

**DESCRITORES**

Classificação; Síndrome Respiratória Aguda Grave; Enfermagem Pediátrica; Recursos Humanos de Enfermagem no Hospital; Segurança do Paciente.

**DESCRIPTORES**

Clasificación; Síndrome Respiratorio Agudo Grave; Enfermería Pediátrica; Personal de Enfermería en Hospital; Seguridad del paciente.

